# Lifestyle and Risk Factors in Hemorrhoidal Disease

**DOI:** 10.3389/fsurg.2021.729166

**Published:** 2021-08-18

**Authors:** Stefania De Marco, Domenico Tiso

**Affiliations:** ^1^Medical Department, Agave Srl, Bologna, Italy; ^2^Department of Nutrition, Accredited Hospital “Villa Maria, ” Rimini, Italy

**Keywords:** Body Mass Index, constipation, lifestyle, conservative treatment, hemorrhoidal disease

## Abstract

Constipation, a low fiber diet, a high Body Mass Index, pregnancy, and a sedentary lifestyle are often assumed to increase the risk of hemorrhoidal disease (HD). However, evidence regarding these factors is controversial. This mini-review aims to examine and critically analyze the association between main risk factors and the prevalence of HD, focusing both on the patient's clinical history and on a tailored treatment. Moreover, some practical suggestions about lifestyle and conservative approaches are given to help clinicians in the management of patients with HD and to obtain the best results from therapy.

## Introduction

Hemorrhoidal disease (HD) has been described in depth throughout medical history. The first manuscript dates back to 37 AD in *De Medicina*, a treatise written by the Roman encyclopedist and physician Aulo Cornelio Celso. The seventh book of *De Medicina* deals with proctological disease: fissure, HD, constipation, and condylomas. The author suggests that manual intervention (surgery) should be taken into consideration when medical therapy has proved ineffective. Moreover, he recommends always integrating medical therapy with a healthy diet and lifestyle. Today, according to the most recent guidelines, conservative therapy is still considered “an effective first-line treatment that should be recommended before surgery” (1).

From the first century AD to 2021, much scientific literature has been published concerning proctological diseases, so much so that HD is among the best described diseases in clinical medicine (2). PubMed today shows 10,016 results when searching the term “Hemorrhoids.” Most of these publications concern surgery and medical treatments. On the contrary, scientific evidence regarding lifestyle and risk factors in HD are limited.

HD is hypothesized to result from deterioration of the supporting connective tissue, prolapse of hemorrhoidal tissue, distention of the hemorrhoidal arteriovenous anastomoses, or dilation of the veins of the internal hemorrhoidal plexus (3–6). Factors commonly assumed to increase the risk of HD include constipation, a low fiber diet, a high Body Mass Index, pregnancy, and a sedentary lifestyle. All these factors need to be more investigated.

This mini-review has been performed in PubMed to identify and summarize the most recent randomized controlled trials (RCTs), meta-analyses, and retrospective and prospective studies that analyze the above-mentioned risk factors involved in HD. The review further aims to give some practical suggestions addressed to reduce the prevalence of the pathology and to alleviate patients' symptoms in combination with the medical treatment.

## Risk Factors

HD can present with a variety of symptoms, including anal bleeding, prolapse, itching, and/or perianal skin irritation. All of these physical discomforts can significantly influence the quality of life (QoL) in patients with HD (2). In addition, frequent recurrence and persisting pain and not negligible complication rate even after surgery (7) raise the need to prevent HD through effective management of risk factors.

## High Body Mass Index

A study published by Riss et al. investigated the prevalence of HD and associated risk factors in an adult general population. The researcher analyzed the correlation between HD and the Body Mass Index (BMI). Of 976 participants enrolled in this prospective study, 380 patients (38.93%) suffered from HD. Among these, 170 patients (44.74%) complained about symptoms associated with HD, whereas 210 patients (55.26%) reported no symptoms. Researchers have observed that the Body Mass Index (BMI) had a significant effect on the occurrence of HD: an increase in BMI increased the risk of HD by 3.5% (8).

Although the correlation between obesity and HD are not plain elucidated, some pathophysiologic mechanisms such as increased intra-abdominal pressure, venous congestion, and chronic inflammation have been hypothesized to contribute to HD development in the obese patients.

On the contrary, Peery et al. in their investigation of 1,074 patients with HD found no correlation with overweight or frankly obese (4).

Studies concerning the correlation of BMI and HD are controversial. The reason is because BMI value is an anthropometric measurement used for categorizing the population, not for identifying visceral fat and its associated risks such as low-grade inflammation. In this respect Gutin observed that: “the tendency toward standardization of obesity measurement and link between body weight and health is in contrast with BMI's ability to accurately diagnose obesity in individuals or populations” (9). Therefore, BMI will continue to be the primary measure given its ease of use and low cost of collection, but it is not the most suitable parameter for measuring individuals' health.

To improve the management and health of patients with HD, it is recommended to routinely evaluate the waist circumference, a useful parameter for predicting low-grade inflammation and associated risks (intra-abdominal pressure and venous congestion). The waist circumference threshold values indicating increased health risk within each BMI category are reported in Table 1 (10).

**Table 1:** Waist circumference thresholds stratified by BMI.

**BMI category (kg/m^**2**^)**	**Waist circumference (cm)**	
	**Women**	**Men**
Normal weight (18.5–24.9)	≥80	≥90
Overweight (25–29.9)	≥90	≥100
Obese I (30–34.9)	≥105	≥110
Obese II and III (≥35)	≥115	≥125

*Waist circumference threshold indicating increased health risk within each BMI category. Data were originally presented in Ross et al. (10)*.

## Constipation

Constipation, hard and dry stool, can worsen symptoms related to hemorrhoidal prolapse. Peery et al. in their study analyzed 1,074 patients with HD and found that constipation, straining during bowel movements and hard or lumpy stools for at least 25% of the time were all associated with an increased prevalence of HD (4). These data were confirmed by Riss et al., who published a cross-sectional study of 976 participants who had undergone a colonoscopy and found that constipation was associated with an increased risk of HD (8).

Therefore, a balanced diet with adequate fiber and fluid intake to improve of stool consistency should be one of the main purposes in conservative treatment for HD (Level of evidence: 1; Grade of recommendation: B) (1).

Alonso-Coello in his meta-analysis (11) show the results of seven randomized trials (378 patients).

Studies with multiple follow-ups, usually at 6 weeks and then at 3 months, showed consistent results over time:

- The risk of not improving/persisting symptoms decreased by 47% in the fiber group (RR = 0.53, 95% CI 0.38–0.73);
- The risk of bleeding decreased by 50% (RR = 0.50, 95% CI 0.28–0.89);
- One study suggested a decrease in recurrence.

As regards the health benefits (laxative effects) attributable to dietary fibers, it is important to analyze in depth the physical effects of insoluble and soluble fiber in the gut (12):

- Insoluble fiber particles (e.g., wheat bran) stimulate secretion of water and mucous by a mechanically irritating effect on large bowel mucosa;
- Soluble gel-forming fiber (e.g., psyllium) has a high capacity to hold water that resists dehydration in the large bowel.

Patients should be advised to avoid straining at stool and to improve bowel function by increasing the intake of soluble fiber, which can increase the volume and improve the softness of fecal mass. HD symptoms may be eased though a regular defecation with type 3 or 4 stool according to the Bristol Stool Form Scale (11, 13).

McRorie et al., in their review, investigated the presence of meaningful clinical evidence in terms of the beneficial effects of different fiber supplements. Among them psyllium, a non-fermented gel-forming fiber, was demonstrated to provide a dichotomous stool normalizing effect: it softens hard stool in constipation and firms up liquid stools in diarrhea, showing to be effective in several clinical studies for constipation treatments (14).

Peery et al. found that fiber intake was associated with a reduced risk of HD. Surprisingly, the association between high fiber intake and reduced risk of HD was held even after adjustment for constipation (4).

Furthermore, recent studies have demonstrated a causal relationship between constipation, dysbiosis, and intestinal peristalsis. Cao et al. in their study suggest that gut dysbiosis could inhibit intestinal motility and contribute to the development and persistence of constipation. The author provides a point of view to demonstrate the pathogenesis of constipation as well as hypothesizing the need for innovative microbiota-mediated therapy for treating chronic constipation (15).

According to the most recent literature, patients suffering from constipation should be treated with a multi-target therapy that acts both on the volume and softness of fecal mass (soluble fiber) and also on gut motility and microbiota (16).

## Sedentary Lifestyle

Data concerning physical activity and HD are controversial: although a sedentary lifestyle is considered a risk factor for developing HD, Perry et al. found an association between a sedentary behavior and a reduced risk, unlike physical activity (4). To better understand this result, it is necessary to analyze the different kinds of sports activity; certain types of exercise can make the problem worse, so it is important to be prudent when choosing how to perform exercise.

The goal of physical activity for people with HD is to promote regular bowel movements, improve circulation, and strengthen muscles in the pelvic area and lower back. On the contrary, a lack of physical activity can contribute to constipation, worsening a current HD, triggering a recurrence, or even causing new problems in those who have never had a hemorrhoid before.

Exercises that are generally considered safe and effective for HD management and prevention include aerobic activities, such as walking and swimming, or controlled-movement exercises to help strengthen the abdominal and rectal tissues, such as yoga.

Patients with HD should avoid exercises that tend to place pressure on sensitive areas, such as cycling, rowing, horseback riding, or some weightlifting exercises that involve the Valsalva maneuver.

Patients should be advised to not give up on exercise, but to pick the right exercise routine. This keeps the system “regular,” promotes colon health, and may even prevent constipation and gastrointestinal disorders. Taken together, moderate physical activity (20–60 min, 3–5 days per week) should be recommended to patients because it improves QoL and can help to effectively manage hemorrhoid symptoms (17).

## Pregnancy

Pregnancy and spontaneous vaginal delivery are predisposing factors for the development of HD due to the constipation and the reduction of venous outflow due to increased circulatory blood volume, venous relaxing effect of progesterone, and also to enlarged uterus that increases pressure in the rectal veins.

The prevalence of HD is mostly in the last trimester of pregnancy and in the first month after delivery, with about 25–35% of pregnant women suffering from this disease (1).

In terms of etiology, mechanical and hormonal factors have been proposed to explain the relationship (18). Straining during defecation, impairment of defecation habits during pregnancy, decrease in physical activity, and psycho-social stress may also predispose to constipation and HD. Progesterone tends to lower the strength of venous wall muscle, decrease circular and longitudinal smooth muscle contractility, and slow gastrointestinal transit. This inhibition contributes to constipation, which indirectly predisposes one to the development of HD. Moreover, dietary modifications can be implicated: decreased fluid intake and iron supplementation may cause constipation.

For many women, symptoms resolve spontaneously soon after birth.

Pregnant women should be advised that preventive methods help significantly with symptom management: dietary modification with increased bulk, such as fresh fruit and vegetables and plenty of water, should be applied during pregnancy and in the postpartum period.

Avoidance of constipation is the most important method for prevention of HD during pregnancy.

## Management of Risk Factors

When intervention for risk factors is not enough to improve the symptoms of patients with HD, many other options are available, ranging from simple conservative measures to surgical excision of the hemorrhoids. The choice of therapy normally depends on the severity of symptoms and the amount of prolapsing hemorrhoidal tissue.

Conservative treatments comprise modern drugs and traditional medicine, available in a variety of formulations, including pills, suppositories, creams, and wipes. Among these, phlebotonics (flavonoids or synthetic compounds such as calcium dobesilate) are widely used for the control of symptoms (Level of evidence: 1; Grade of recommendation: B) (1); they are able to improve vascular tone, decrease capillary permeability, reduce venous capacity, and facilitate lymphatic drainage, as well as having anti-inflammatory effects (19).

Oral and topical therapies are largely used for the treatment of low-grade hemorrhoids, but unfortunately these treatments have met with criticism. As regards pills and oral delivery, some physiological mechanisms can reduce intestinal absorption of the active ingredients of the drugs. The absorption by the mucous secretions and the cytochrome CYP3A and P450 activity can make the active principles unavailable. Moreover, the peptide nature of the compounds can cause their gastric hydrolysis, and the saprophytic flora can cause a reduced intestinal absorption of the active ingredients. Consequently, only a small percentage of active compounds is actually absorbed, requiring high doses of treatment to reach the therapeutic level (20). An example could be represented by the flavonoid diosmin: the molecular size impacts on the extent of absorption, and it needs to be micronized to have better clinical efficacy (21).

**Figure 1:**
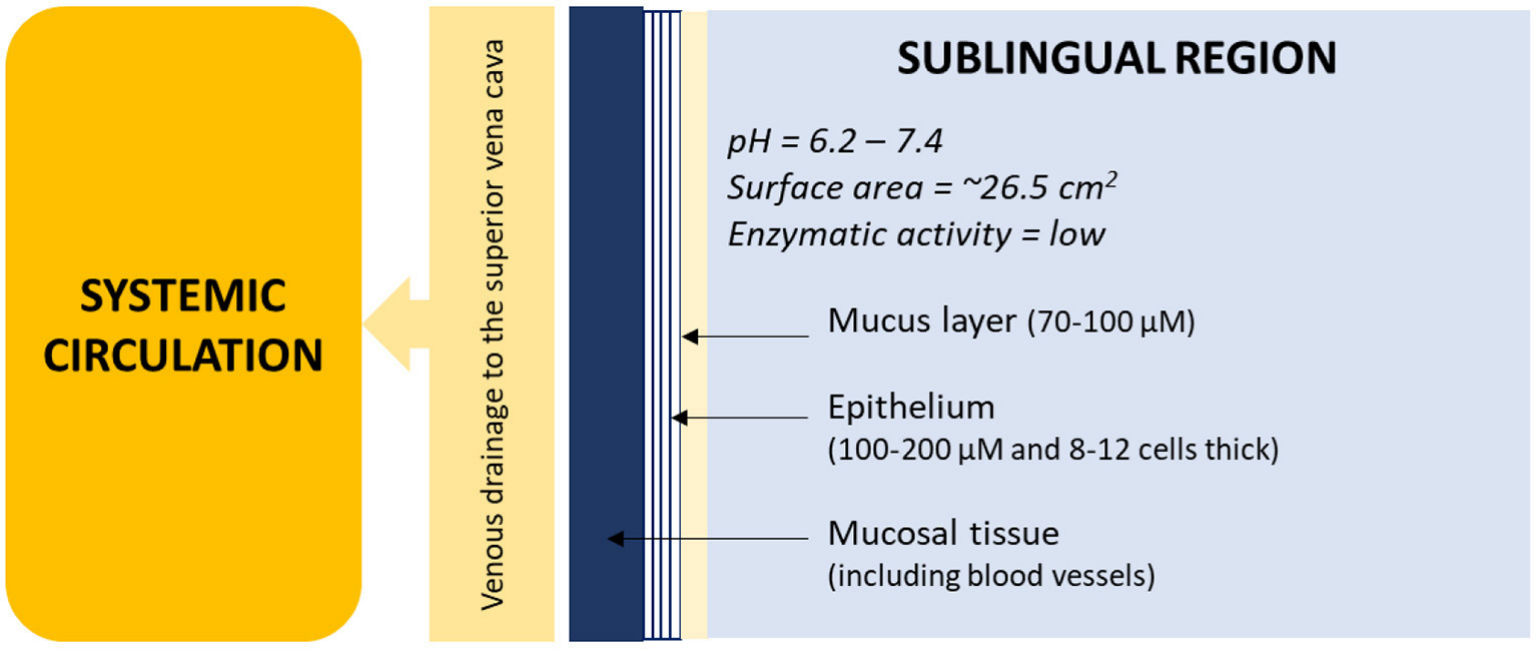
Schematic diagram of the sublingual regions in the oral cavity. Data were originally presented in Hua (22).

To further improve the pharmacokinetics of this drug, a solution could be a sublingual formulation. In fact, among the oral administrations, the sublingual route of administration has significant advantages for systemic drug delivery. Drugs can be quickly and directly absorbed into the systemic circulation *via* venous drainage to the superior vena cava. Therefore, sublingual administration is particularly functional for drugs that undergo high hepatic clearance or degradation in the gastrointestinal tract and for patients with swallowing problems (Figure 1) (22).

The treatment of HD remains challenging. It involves the degeneration of the supporting tissue of the anal cushions, venous dilation, blood stagnation, the formation of edematous venous plexus, and inflammation. Multiple treatment options are available, but patients rightly demand a tailored and effective approach.

Considering the complexity of the pathophysiology of HD, the treatment option should be oriented to a multi-target treatment capable of acting on all pathological mechanisms simultaneously. Furthermore, a pharmaceutical form with high bioavailability should be preferred.

Among the conservative therapy for HD, topical treatments are used as first-line treatment and as a bridge to surgery. The primary objective of most topical treatment aims to manage the symptoms rather than to cure them. These topical medications can contain different ingredients, such as corticosteroids, antiseptics and anesthetics (23). The main concerns of these topical treatments concern their prolonged application that can induce sensitization reactions, immunosuppression, and vessel reactivity (due to cortisone) (24), irritation, and resistance (due to lidocaine).

An improvement of this kind of treatment can be represented by an anorectal gel based on natural ingredients with film-forming and protective actions. An innovative formulation should have an approach that considers all aspects of HD; it should hydrate and offer immediate comfort by a lubricating activity and, simultaneously, restore the function of the skin barrier and of the tissue repair processes and support the connective tissue stability whose reduction has been associated with the incidence of HD (6).

## Conclusions

The therapeutic treatment of hemorrhoids ranges from dietary and lifestyle modification to radical surgery, depending on the degree and severity of symptoms.

Body weight has been often correlated to an increased risk for HD, but data obtained from clinical studies about the association of high BMI (>25) and HD are controversial. This because HD in the obese is not directly and exclusively associated with one's weight, but to intra-abdominal pressure, venous congestion, and the chronic inflammation (9). Hence, the right indicator for measuring the health status of patients is rather the waist circumference, instead of BMI.

In clinical studies of HD, dietary fiber supplements resulted in an effective treatment in non-prolapsing hemorrhoids, reducing the risk of persisting symptoms and bleeding by ~50% (25). As fiber supplements are safe and useful, they can represent a first treatment or an integration of other therapeutic modalities of HD. However, fiber supplements could take up to 6 weeks for a significant improvement (26), taking into consideration the need to choose the appropriate fiber and for it to simultaneously act on the intestinal motility and gut microbiota (15).

Beyond the increase in the intake of dietary soluble fiber, lifestyle modification should also be advised to any patient with any degree of hemorrhoids. These changes include adequate fluid intake, regular exercise, improving anal hygiene, avoiding straining at stool, and, when necessary, integrating these with appropriate and tailored treatments.

In order to positively affect the QoL of patients with HD, it is important to analyze the above-mentioned risk factors, remembering that a good analysis starts from good listening.

Several studies have highlighted the importance of good interactions between physicians and patients. The evidence shows that by allowing the patients to discuss their problem without being interrupted for first two minutes, it is possible to optimize the visit time and obtain better results (27, 28).

Skillful listening is essential to make accurate diagnoses, to educate patients on the culture of prevention, and to convey empathy and support. Reporting a quote from Hippocrates, the father of medicine, 2,500 years ago: “It is more important to know what sort of person has a disease than to know what sort of disease a person has”.

## Author Contributions

DT conceived and supervised the review topics. SDM wrote the first draft. All authors contributed to the article and approved the submitted version.

## Conflict of Interest

This work received funding from Agave SR, Italy. The funder had the following involvement with the work: SDM was employed by Agave SRL, Italy; DT is a consultant Agave SRL, Italy.

## Publisher's Note

All claims expressed in this article are solely those of the authors and do not necessarily represent those of their affiliated organizations, or those of the publisher, the editors and the reviewers. Any product that may be evaluated in this article, or claim that may be made by its manufacturer, is not guaranteed or endorsed by the publisher.

## Abbreviations

QoL: Quality of Life
HD: Hemorrhoidal disease
BMI: Body Mass Index
RR: Relative Risk
CI: Confidence Interval.
